# Prevalence of SARS-CoV-2 infection and impact of the COVID-19 pandemic in avocado farmworkers from Mexico

**DOI:** 10.3389/fpubh.2023.1252530

**Published:** 2023-12-20

**Authors:** Cynthia Armendáriz-Arnez, Marcela Tamayo-Ortiz, Francisco Mora-Ardila, María Esther Rodríguez-Barrena, David Barros-Sierra, Federico Castillo, Armando Sánchez-Vargas, David Lopez-Carr, Julianna Deardorff, Brenda Eskenazi, Ana M. Mora

**Affiliations:** ^1^Escuela Nacional de Estudios Superiores Unidad Morelia, Universidad Nacional Autónoma de México, Morelia, Mexico; ^2^Department of Environmental Health Sciences, Columbia Mailman School of Public Health, New York, NY, United States; ^3^Instituto de Investigaciones en Ecosistemas y Sustentabilidad, Universidad Nacional Autónoma de México, Morelia, Mexico; ^4^Instituto Mexicano del Seguro Social, Mexico City, Mexico; ^5^Department of Environmental Science, Policy and Management, University of California, Berkeley, Berkeley, CA, United States; ^6^Institute of Economic Research, Universidad Nacional Autónoma de Mexico, Mexico City, Mexico; ^7^Department of Geography, University of California, Santa Barbara, Santa Barbara, CA, United States; ^8^Center for Environmental Research and Community Health, School of Public Health, University of California, Berkeley, Berkeley, CA, United States

**Keywords:** farmworker health, anxiety, depression, food insecurity, COVID-19, Mexico

## Abstract

**Introduction:**

The COVID-19 pandemic disproportionately affected farmworkers in the United States and Europe, leading to increased morbidity and mortality. However, little is known about the specific impact of the pandemic on agriculture and food production workers in low- and middle-income countries. This study aimed to investigate the prevalence of SARS-CoV-2 infection and assess the mental health and economic consequences of the COVID-19 pandemic among avocado farmworkers in Michoacan, Mexico.

**Methods:**

We conducted a cross-sectional study of adult farmworkers (*n* = 395) in May 2021. We collected survey data, nasal swabs and saliva samples for SARS-CoV-2 RNA detection, and blood samples for immunoglobulin G (IgG) reactivity measurements.

**Results:**

None of the farmworkers tested positive for SARS-CoV-2 RNA. However, among unvaccinated farmworkers (*n* = 336, 85%), approximately one-third (33%) showed evidence of past infection (positive for IgG against SARS-CoV-2). Unvaccinated farmworkers who lived with other farmworkers (aRR = 1.55; 95% CI: 1.05, 2.05), had ever lived with someone with COVID-19 (aRR = 1.82; 95% CI: 1.22, 2.43), and who had diabetes (aRR = 2.19; 95% CI: 1.53, 2.85) had a higher risk of testing IgG-positive for SARS-CoV-2 infection. In contrast, unvaccinated farmworkers living in more rural areas (outside of Tingambato or Uruapan) (aRR = 0.71; 95% CI: 0.46, 0.96) or cooking with wood-burning stove (aRR = 0.75; 95% CI: 0.55, 0.96) had a lower risk of IgG-positivity. Moreover, 66% of farmworkers reported a negative impact of the pandemic on their lives, 29% reported experiencing food insecurity and difficulty paying bills, and 10% reported depression or anxiety symptoms.

**Conclusion:**

The COVID-19 pandemic has significantly affected the mental health and financial well-being of avocado farmworkers. Consequently, the implementation of interventions and prevention efforts, such as providing mental health support and food assistance services, is imperative.

## Introduction

The first confirmed case of COVID-19 in Mexico was reported on February 27, 2020 (1). About a month later, when the number of confirmed cases in the country had surged to 848 (2), the Mexican federal government launched the “National Healthy Distance” campaign (3). This campaign urged people to wash their hands frequently, practice social distancing, and, if possible, work from home. On March 30, 2020, the federal government declared a national health emergency and suspended all non-essential activities (4). Farmworkers were among the occupational groups deemed essential to the economy (5) and continued in-person work. On June 1, 2020, with no clear signs that transmission had been brought under control, the federal government introduced a “traffic light” alert system for epidemiological risk in each state, guiding state responses and replacing nationwide suspensions (6). In August 2020, Mexico ranked among the top 10 countries in terms of infections and the top five in terms of deaths per 100,000 inhabitants (7). The COVID-19 vaccination campaign, led by the Mexican federal government, began on December 24, 2020, prioritizing healthcare workers, teachers, and adults aged over 60 years (8).

Approximately 21% of Mexico’s population resides in rural areas (9), with 12% employed in the agricultural sector (10). Both farmworkers and non-farmworkers living in rural areas face similar poverty indicators, such as educational attainment, income level, food access, housing quality, and healthcare accessibility (11). The COVID-19 pandemic has exacerbated long-standing socioeconomic and health disparities in historically underserved populations in Mexico (12–15) and worldwide (16, 17), including those living in rural areas. One of the most glaring disparities highlighted by the COVID-19 pandemic was the limited access to healthcare in rural areas, where about one in four people has access to adequate facilities (18). This lack of healthcare infrastructure made it exceedingly difficult for individuals in the rural areas to receive prompt medical attention and SARS-CoV-2 testing, thereby exacerbating virus spread and increasing the risk of severe illness or death. Moreover, poverty remains more prevalent in rural areas (19), introducing additional risk factors for SARS-CoV-2 infection like malnutrition and overcrowded housing. Despite the availability of COVID-19 vaccines to non-healthcare professionals in Mexico since February 2021, vaccine hesitancy is slightly more prevalent among rural and indigenous communities (20). This hesitancy often stems from limited access to reliable information, historical distrust of healthcare systems, and misinformation, contributing to lower vaccination rates in these populations (21).

Epidemiological studies conducted in the United States (22–26) and Europe (27, 28) have shown that farmworkers are among the populations disproportionately affected by the COVID-19 pandemic. However, little is known about the burden among farmworkers in Mexico and other low- and middle- income countries (LMICs). Recently, a study of banana farmworkers in Guatemala conducted from June 2020 to October 2021 (29) found a SARS-CoV-2 infection prevalence (3.1 cases/100 person-years) similar to the one reported in a study of primarily Mexican-born and low-income farmworkers from California conducted from July to November 2020 (22). The latter study found that some of the risk factors for SARS-CoV-2 infection among farmworkers were low educational attainment, living in crowded housing or with unrelated roommates, living in urban areas, and working in the fields (or outdoors) rather than elsewhere in agriculture (30). Farmworkers in Mexico, while having different living and working conditions from those in the U.S. and Europe, likely share commonalities in various structural factors and social determinants of health, such as limited healthcare access, economic vulnerability, and labor rights (31, 32). These shared factors could potentially increase Mexican farmworkers’ risk for SARS-CoV-2 infection and the negative impact of the COVID-19 pandemic on their lives. Notably, the study of farmworkers in Guatemala observed that those who experienced COVID-19 had greater disease severity, absenteeism, and economic losses than farmworkers with other influenza-like illnesses (29). Likewise, recent cross-sectional studies of primarily Mexican-born farmworkers in California and Washington State found that the COVID-19 pandemic exacerbated challenges affecting mental health and food security among this vulnerable population (33–35).

Mexico is the world’s largest producer of avocados (36), accounting for 45% of global production (37). As of 2022, the state of Michoacan produced approximately 72% of the nation’s avocados (37) and was home to around 34,000 avocado farmers (38). Michoacan has a population of 4.7 million people, with about 30% residing in rural areas (39). The state has played a crucial role in the dynamics of agricultural labor migration to the U.S. (40). For instance, between 2015 and 2020, Michoacan ranked third among the 32 Mexican states in the number of immigrants to the United States, with nearly 40,000 immigrants (41). Due to the state’s heavy reliance on agriculture and its substantial rural population, understanding the impact of the COVID-19 pandemic on its residents, especially those employed in the avocado industry, holds significant importance. This study aimed to assess the prevalence of SARS-CoV-2 infection among Michoacan avocado farmworkers in May 2021. Additionally, it examined sociodemographic, household, community, and workplace factors associated with prior SARS-CoV-2 infection (as indicated by immunoglobulin G (IgG) seropositivity) among unvaccinated farmworkers. Lastly, the study delved into evaluating the mental health and economic impact of the COVID-19 pandemic on both unvaccinated and vaccinated farmworkers.

## Methods

### Study setting

Tingambato is a rural community located within the state of Michoacan, Mexico. It has a population of approximately 16,000 inhabitants, which accounts for 0.3% of the state’s population. The population density in Tingambato is 86.0 people per square kilometer (42). The community is also home to more than 2,500 registered avocado growers (43). Uruapan del Progreso, on the other hand, is the second largest city in Michoacan, with around 360,000 inhabitants and a population density of 352.2 people per square kilometer (42); it is situated 30 kilometers away from Tingambato. Tingambato and Uruapan differ in several aspects, including size, infrastructure, cultural significance, delinquency rates, and economic activities (44–46). Notably, people from Tingambato and 10 neighboring towns heavily depend on services and infrastructure provided by the city of Uruapan, including healthcare facilities, educational institutions, and transportation (44).

### Study procedures

This study was conducted in partnership with the Tingambato Local Plant Health Board (LPHB), an organization of avocado growers that monitors local farms’ compliance with the Good Agricultural Practices program (47). The study was approved by the Institutional Review Boards at UC Berkeley and Escuela Nacional de Estudios Superiores Unidad Morelia, Universidad Nacional Autónoma de México (UNAM).

In April and May 2021, we invited farmworkers from avocado farms registered at the Tingambato LPHB to participate in our study. We advertised the study on local radio, through community groups and growers, and via flyers posted around town. Farmworkers were eligible for participation if they were 18 years or older, not pregnant, and had worked at an avocado farm within the previous 2 weeks. We enrolled a convenience sample of 400 farmworkers from May 17 to May 29, 2021, during a period of low incidence of cases of COVID-19 after the January 2021 wave (Supplementary Figure S1) and at the time when only individuals who were 50 years old or older were eligible to be vaccinated against SARS-CoV-2 (48, 49). We excluded from analyses 5 (1.3%) farmworkers who did not provide blood samples, leaving a total of 395 participants.

All participants completed an in-person visit at the LPHB offices. Upon providing written informed consent, participants independently completed a 45-min computer-based questionnaire in Spanish, comprising approximately 300 questions (available upon request). The questionnaire encompassed two primary components: one focusing on COVID-19 and the other on pesticide exposure and its health effects among farmworkers. It gathered information on sociodemographic, household, community, and workplace characteristics; COVID-19 related symptoms (i.e., cough, blocked or runny nose, fever or chills, headache, sore throat, myalgia or body aches, shortness or difficulty breathing, diarrhea, nausea, fatigue, and new loss of sense of taste or smell) experienced since the pandemic started in December 2019; number of test-confirmed SARS-CoV-2 infections; COVID-19 exposures and vaccination status; and consequences of the COVID-19 pandemic on daily life and well-being. Regarding vaccination status, a farmworker was considered fully vaccinated if she/he had received all recommended doses of a COVID-19 vaccine authorized or approved by the Mexican Federal Commission for Protection against Sanitary Risks (COFEPRIS) or listed for emergency use by the World Health Organization (WHO). Partially vaccinated farmworkers had received at least one dose but had not completed all recommended doses of the vaccine. For analysis purposes, partially and fully vaccinated individuals were combined into one category because most vaccinated farmworkers (70%) did not specify whether they had received one or both doses of the COVID-19 vaccine.

To understand participants’ own assessment of the impact of the pandemic, we asked them two questions: “How much of a negative impact has the COVID-19 pandemic had on your life?” and “How concerned are you about COVID-19?” We also asked study participants whether they had increased their use of alcohol, tobacco, marijuana, and other substances like pills or other drugs “compared to their use before the COVID-19 pandemic.” We asked study participants about changes in other behaviors, such as less physical activity and sleeping problems during this time frame. To ascertain symptoms of depression and anxiety in the 2 weeks preceding the interview, we used the Patient Health Questionnaire-2 (PHQ-2) (50) and the Generalized Anxiety Disorder-2 (GAD-2) scale (51), respectively, and classified participants with scores ≥2 on either scale as symptomatic. To assess household food insecurity, we adapted the U.S. Department of Agriculture (USDA) Household food security six-question survey (52) by altering the time period to “since the pandemic started in Mexico in March 2020″ rather than “the last 12 months.” For analyses, levels of food insecurity defined using USDA cut-offs were collapsed into two categories: the two lowest food insecurity groups (low and very low) were classified as “not experiencing food insecurity” and the two highest groups (high and marginal) were classified as “experiencing food insecurity.” In addition, we asked participants the question: “Have you had more difficulty paying your bills (water, gas and electricity, rent) since the pandemic started?.” We asked participants who were receiving remittances from family members outside of Mexico just prior to the pandemic whether they were now receiving less, more, or the same.

After completing the questionnaire, trained research staff measured participants’ height and weight to calculate their body mass index (BMI). They also collected a nasal swab and a saliva sample for detection of SARS-CoV-2 RNA via rapid SARS-CoV-2 antigen test (Panbio COVID-19 Rapid Device) and real-time reverse transcriptase polymerase chain reaction (RT-PCR) assay (53), respectively. Lastly, a licensed phlebotomist collected a 4-mL non-fasting blood sample via venipuncture for assessment of IgG reactivity against the SARS-CoV-2 spike protein (which does not discriminate between natural SARS-CoV-2 infection and vaccination status) via chemiluminescent microparticle immunoassay (54). Nasal swabs were tested on the spot; saliva and blood specimens were stored at 4°C and − 20°C, respectively, until shipment to the National Institute of Genomic Medicine in Mexico City for analysis.

### Statistical analyses

#### Prevalence and risk factors for positive SARS-CoV-2 infection test results

We tabulated SARS-CoV-2 RNA and IgG test results for all farmworkers and then computed estimates of the proportion of positive tests by vaccination status.

No farmworkers had nasal swabs or saliva samples positive for SARS-CoV-2 RNA, so we were not able to examine risk factors associated with positivity on these tests. Furthermore, due to our inability to discriminate between farmworkers who were vaccinated and those who had a SARS-CoV-2 infection, as well as the sociodemographic differences between vaccinated and unvaccinated participants (Table 1), we restricted our analyses on risk factors associated with IgG-positive results to unvaccinated farmworkers (*n* = 336). First, we fitted univariate binomial logistic regression models to examine the association of a wide range of sociodemographic, household, community, and work-related characteristics (referred to henceforth as risk factors) with IgG status (positive/negative) (Table 2). Categorical risk factors were modeled as shown in Table 2; age and household size were modeled as continuous variables. We then fitted a multivariable binomial logistic regression model with all risk factors with *p* < 0.20 in bivariate analyses. We used multiple imputation with chained equations (10 iterations) to account for missing values in our multivariable model. We reduced our model using an information approach (55) and the *MuMIn* package; only risk factors with an importance value ≥0.5 in at least one of the 10 iterations were retained. Lastly, we estimated adjusted relative risks (aRRs) from our reduced model using the *logisticRR* package (56).

**Table 1:** Characteristics of avocado farmworkers, Michoacan, Mexico, May 2021 [*n* (%), median (IQR), or mean ± SD].

Attribute	All (*n* = 395)	Unvaccinated (*n* = 336)^a^	Vaccinated (*n* = 56)
Sociodemographic characteristics			
Sex			
Female	9 (2.3)	9 (2.7)	0 (0.0)
Male	386 (97.7)	327 (97.3)	56 (100.0)
Age (years)			
Median (IQR)	37 (28–48)	35 (27–45)	60 (44–65)
<30	123 (31.2)	116 (34.5)	4 (7.1)
30–59	228 (57.7)	206 (61.3)	22 (39.3)
≥60	42 (10.6)	12 (3.6)	30 (53.6)
No answer	2 (0.5)	2 (0.6)	0 (0.0)
Education			
≤Primary school	108 (27.3)	95 (28.3)	13 (23.2)
Middle school completed	115 (29.1)	106 (31.5)	8 (14.3)
High school completed	81 (20.5)	74 (22.0)	5 (8.9)
Bachelor’s degree or technical studies completed	74 (18.7)	46 (13.7)	28 (50.0)
No answer	17 (4.3)	15 (4.5)	2 (3.6)
Marital status			
Not married or living as married	91 (23.0)	82 (24.4)	7 (12.5)
Married or living as married	302 (76.5)	252 (75.0)	49 (87.5)
No answer	2 (0.5)	2 (0.6)	0 (0.0)
Community of residence			
Tingambato	242 (61.3)	199 (59.3)	42 (75.0)
Uruapan	38 (9.6)	26 (7.7)	11 (19.6)
Other town^b^	115 (29.1)	111 (33.0)	3 (5.4)
Household characteristics			
Household size			
Median (IQR)	3 (2–5)	3 (2–5)	4 (2–5)
0–3	103 (26.1)	87 (25.9)	16 (28.6)
3–4	94 (23.8)	83 (24.7)	10 (17.9)
4–13	185 (46.8)	156 (46.4)	27 (48.2)
No answer	13 (3.3)	10 (3.0)	3 (5.4)
Household crowding			
≤2 persons per bedroom	333 (84.3)	281 (83.6)	49 (87.5)
>2 persons per bedroom	41 (10.4)	37 (11.0)	4 (7.1)
No answer	21 (5.3)	18 (5.4)	3 (5.4)
Living with other farmworkers			
No	175 (44.3)	152 (45.2)	22 (39.3)
Yes	210 (53.2)	176 (52.4)	32 (57.1)
No answer	10 (2.5)	8 (2.4)	2 (3.6)
Annual household income (USD $)			
≤3,000	120 (30.4)	107 (31.8)	11 (19.6)
>3,000-6,000	143 (36.2)	126 (37.5)	17 (30.4)
>6,000	61 (15.4)	42 (12.5)	19 (33.9)
No answer	71 (18.0)	61 (18.2)	9 (16.1)
Occupational characteristics			
Job title			
Owner	88 (22.3)	68 (20.2)	19 (33.9)
Full-time worker	137 (34.7)	132 (39.3)	5 (8.9)
Temporary worker^c^	12 (3.0)	12 (3.6)	0 (0.0)
No answer^d^	158 (40.0)	124 (36.9)	32 (57.1)
Type of agricultural work in the last 2 weeks^e^			
Land preparation (watering, sowing, soil mix)	252 (63.8)	210 (62.5)	40 (71.4)
Pesticide application	324 (82.0)	271 (80.7)	52 (92.9)
Crop maintenance (pruning, weeding)	324 (82.0)	273 (81.2)	51 (91.1)
Cleaning storage room or packing fruit	243 (61.5)	202 (60.1)	41 (73.2)
Worker supervision	129 (32.7)	109 (32.4)	20 (35.7)
No answer	5 (1.3)	5 (1.5)	0 (0.0)
Working closely with other farmworkers			
No	19 (4.8)	16 (4.7)	1 (1.8)
Yes	371 (93.9)	315 (93.8)	55 (98.2)
No answer	5 (1.3)	5 (1.5)	0 (0.0)
Health-related characteristics			
Body mass index (measured)			
Mean ± SD	28.5 ± 4.4	28.6 ± 4.6	28.01 ± 3.44
Underweight or normal (<25)	88 (22.3)	74 (22.0)	13 (23.2)
Overweight (25–30)	175 (44.3)	148 (44.0)	25 (44.6)
Obesity (≥30)	131 (33.2)	113 (33.6)	18 (32.1)
Not collected	1 (0.3)	1 (0.3)	0 (0.0)
Self-reported diabetes			
No	374 (94.7)	325 (96.7)	46 (82.1)
Yes	21 (5.3)	11 (3.3)	10 (17.9)
Self-reported hypertension			
No	351 (88.9)	306 (91.1)	42 (75.0)
Yes	44 (11.1)	30 (8.9)	14 (25.0)
Health insurance coverage			
None	199 (50.4)	184 (54.8)	12 (21.4)
IMSS	103 (26.1)	90 (26.8)	13 (23.2)
ISSSTE	47 (11.9)	17 (5.1)	30 (53.6)
Other	46 (11.6)	45 (13.4)	1 (1.8)
Any COVID-19 related symptoms since the pandemic started^f^			
No	156 (39.5)	131 (39.0)	22 (39.3)
Yes	239 (60.5)	205 (61.0)	34 (60.7)
Self-reported positive COVID-19 test			
Never	377 (95.4)	322 (96.4)	52 (94.5)
Ever	15 (3.8)	12 (3.6)	3 (5.5)
No answer	3 (0.8)	2 (0.6)	1 (1.8)

IMSS, Instituto Mexicano de Seguridad Social (Mexican Social Security Institute); ISSSTE, Instituto de Seguridad y Servicios Sociales de los Trabajadores del Estado (Mexican Institute for Social Security and Services for State Workers); IQR, interquartile range. ^a^Three participants did not answer whether they were vaccinated or not. ^b^This category includes the following towns (distance to Tingambato): La Escondida (28.4 km), El Mesón (22.9 km), Angachuan (37.6 km), Pichátaro (55.4 km), Parangüitiro (15 km), Ziracuaretiro (18 km), San Angel Zurumucapio (23 km), Nahuatzen (45 km), Comachuén (45.2 km), Tancítaro (56.1 km), El Tarascón (Salvador Escalante 31.7 km), Nocutzepo (Erongarícuaro 49.2 km), Pátzcuaro (57.4 km). ^c^This category includes workers who are hired for specific activities such as pesticide application and harvest. ^d^There was a programming mistake in the computer-based questionnaire that caused all participants who reported working in only one farm to skip this question. ^e^Some participants worked in a variety of jobs. ^f^Participants were asked whether they had experienced any of the following symptoms since December 2019: fever or chills, cough, shortness of breath, fatigue, myalgia, headache, new loss of sense of smell or taste, sore throat, blocked or runny nose, nausea or vomit, diarrhea, or any other symptom.

**Table 2:** Sociodemographic, household, occupational, and health-related risk factors for anti-SARS-CoV-2 IgG positivity among unvaccinated avocado farmworkers, Michoacan, Mexico, May 2021 [*n* (%), median (IQR), or mean ± SD].

Attribute	All (*n* = 336)	IgG-positivity			
		Yes (*n* = 112)	No (*n* = 224)	*p*-value^a^	aRR (95% CI)^b^
Sociodemographic risk factors					
Sex					
Female	9 (2.7)	4 (44.4)	5 (55.6)	0.48	--
Male	327 (97.3)	108 (33.0)	219 (67.0)		--
Age (years)					
Median (IQR)	35 (27–45)	34.5 (27–46)	34.5 (27–45)		--
<30	116 (34.5)	38 (32.8)	78 (67.2)	0.20	--
30–59	206 (61.3)	67 (32.5)	139 (67.5)		--
≥60	12 (3.6)	7 (58.3)	5 (41.7)		--
No answer	2 (0.6)	0 (0.0)	2 (100.0)		--
Education					
≤Primary school	95 (28.3)	27 (28.4)	68 (71.6)	0.70	--
Middle school completed	106 (31.5)	37 (34.9)	69 (65.1)		--
High school completed	74 (22.0)	25 (33.8)	49 (66.2)		--
Bachelor’s degree or technical studies completed	46 (13.7)	17 (37.0)	29 (63.0)		--
No answer	15 (4.5)	6 (40.0)	9 (60.0)		--
Marital status					
Not married or living as married	82 (24.4)	28 (34.1)	54 (65.9)	0.89	--
Married or living as married	252 (75.0)	84 (33.3)	168 (66.7)		--
No answer	2 (0.6)	0 (0.0)	2 (100.0)		--
Annual household income (USD $)					
3,000	107 (31.8)	31 (29.0)	76 (71.0)	0.36	--
>3,000-6,000	126 (37.5)	37 (29.4)	89 (70.6)		--
>6,000	42 (12.5)	17 (40.5)	25 (59.5)		--
No answer	61 (18.2)	27 (44.3)	34 (55.7)		--
Community of residence					
Tingambato	199 (59.3)	69 (34.7)	130 (65.3)	0.17	Reference
Uruapan	26 (7.7)	12 (46.2)	14 (53.8)		1.17 (0.68, 1.67)
Other town^c^	111 (33.0)	31 (27.9)	80 (72.1)		0.71 (0.46, 0.96)
Household and community risk factors					
Household size					
Median (IQR)	3 (2–5)	4 (3–5)	3 (2–5)		--
0–3	87 (25.9)	24 (27.6)	63 (72.4)	0.41	--
3–4	83 (24.7)	27 (32.5)	56 (67.5)		--
4–13	156 (46.4)	56 (35.9)	100 (64.1)		--
No answer	10 (3.0)	5 (50.0)	5 (50.0)		--
Wood-burning cooking stove					
No^d^	210 (62.5)	81 (38.6)	129 (61.4)	0.01	Reference
Yes	126 (37.5)	31 (24.6)	95 (75.4)		0.75 (0.55, 0.96)
Children <18 y living in the home					
No	66 (19.6)	18 (27.3)	48 (72.7)	0.19	--
Yes	255 (75.9)	91 (35.7)	164 (64.3)		--
No answer	15 (4.5)	3 (20.0)	12 (80.0)		--
Children ≤5 y living in the home					
No	176 (52.4)	53 (30.1)	123 (69.9)	0.08	--
Yes	134 (39.9)	53 (39.6)	81 (60.4)		--
No answer	26 (7.7)	6 (23.1)	20 (76.9)		--
Living with other farmworkers					
No	152 (45.2)	39 (25.7)	113 (74.3)	0.01	Reference
Yes	176 (52.4)	70 (39.8)	106 (60.2)		1.55 (1.05, 2.05)
No answer	8 (2.4)	3 (37.5)	5 (62.5)		--
Household crowding					
≤2 persons per bedroom	281 (83.6)	90 (32.0)	191 (68.0)	0.18	--
>2 persons per bedroom	37 (11.0)	16 (43.2)	21 (56.8)		--
No answer	18 (5.4)	6 (33.3)	12 (66.7)		--
Face covering use all the time					
No	168 (50.0)	63 (37.5)	105 (62.5)	0.31	--
Yes	138 (41.1)	44 (31.9)	94 (68.1)		--
No answer	30 (8.9)	5 (16.7)	25 (83.3)		--
Possible exposure to someone with COVID-19 at home since the start of the pandemic^e^					
No	299 (89.0)	90 (30.1)	209 (69.9)	<0.01	Reference
Yes	20 (6.0)	14 (70.0)	6 (30.0)		1.82 (1.22, 2.43)
No answer	17 (5.1)	8 (47.1)	9 (52.9)		--
Occupational risk factors					
Job title					
Owner	68 (20.2)	20 (29.4)	48 (70.6)	0.80	--
Full-time worker	132 (39.3)	43 (32.6)	89 (67.4)		--
Temporary worker^f^	12 (3.6)	3 (25.0)	9 (75.0)		--
No answer^g^	124 (36.9)	46 (37.1)	78 (62.9)		--
Worked indoors					
No	129 (38.4)	48 (37.2)	81 (62.8)	0.22	--
Yes	202 (60.1)	62 (30.7)	140 (69.3)		--
No answer	5 (1.5)	2 (40.0)	3 (60.0)		--
Face covering use at work all the time					
No	245 (72.9)	80 (32.7)	165 (67.3)	0.57	--
Yes	89 (26.5)	32 (36.0)	57 (64.0)		--
No answer	2 (0.6)	0 (0.0)	2 (100.0)		--
Fever and symptoms screening upon arrival at workplace					
Rarely	180 (53.6)	54 (30.0)	126 (70.0)	0.19	--
Often	118 (35.1)	44 (37.3)	74 (62.7)		--
No answer	38 (11.3)	14 (36.8)	24 (63.2)		--
Possible exposure to someone with COVID-19 at work since the start of the pandemic^h^					
No	276 (82.1)	87 (31.5)	189 (68.5)	0.22	--
Yes	33 (9.8)	14 (42.4)	19 (57.6)		--
No answer	27 (8.0)	11 (40.7)	16 (59.3)		--
Health-related characteristics					
Body mass index (measured)					
Mean ± SD	28.6 ± 4.6	28.5 ± 4.4	28.5 ± 4.6		--
Underweight or normal (<25)	74 (22.0)	26 (35.1)	48 (64.9)	0.57	--
Overweight (25–30)	148 (44.0)	45 (30.4)	103 (69.6)		--
Obesity (≥30)	113 (33.6)	41 (36.3)	72 (63.7)		--
Not collected	1 (0.3)	0 (0.0)	1 (100.0)		--
Self-reported diabetes					
No	325 (96.7)	105 (32.3)	220 (67.7)	0.04	Reference
Yes	11 (3.3)	7 (63.6)	4 (36.4)		2.19 (1.53, 2.85)
Self-reported hypertension					
No	306 (91.1)	104 (34.0)	202 (66.0)	0.41	--
Yes	30 (8.9)	8 (26.7)	22 (73.3)		--
Health insurance coverage					
None	184 (54.8)	65 (35.3)	119 (64.7)	0.56	--
IMSS	90 (26.8)	30 (33.3)	60 (66.7)		--
ISSSTE	17 (5.1)	6 (35.3)	11 (64.7)		--
Other	45 (13.4)	11 (24.4)	34 (75.6)		--
Any COVID-19 related symptoms since the pandemic started^i^					
No	131 (39.0)	38 (29.0)	93 (71.0)	0.18	--
Yes	205 (61.0)	74 (36.1)	131 (63.9)		--
Self-reported positive COVID-19 test					
Never	322 (96.4)	99 (30.7)	223 (69.3)	<0.01	--
Ever	12 (3.6)	12 (100.0)	0 (0.0)		--
No answer	2 (0.6)	1 (50.0)	1 (50.0)		--

aRR, adjusted relative risk; IMSS, Instituto Mexicano de Seguridad Social (Mexican Social Security Institute); ISSSTE, Instituto de Seguridad y Servicios Sociales de los Trabajadores del Estado (Mexican Institute for Social Security and Services for State Workers); IQR, interquartile range. Missing entries were excluded from bivariate analyses. ^a^p-values were calculated using univariate binomial logistic regression models. ^b^Adjusted RR and 95% CI were only obtained for variables with p < 0.20 in bivariate analyses and an importance value ≥ 0.5 in at least one of the 10 iterations in multivariable model. ^c^This category includes the following towns (distance to Tingambato): La Escondida (28.4 km), El Mesón (22.9 km), Angachuan (37.6 km), Pichátaro (55.4 km), Parangüitiro (15 km), Ziracuaretiro (18 km), San Angel Zurumucapio (23 km), Nahuatzen (45 km), Comachuén (45.2 km), Tancítaro (56.1 km), El Tarascón (Salvador Escalante 31.7 km), Nocutzepo (Erongarícuaro 49.2 km), Pátzcuaro (57.4 km). ^d^Includes electric and gas stoves. ^e^Lived with someone who had COVID-19 symptoms or were known to be infected with SARS-CoV-2 since December 2019. ^f^Includes workers who are hired for specific activities such as pesticide application and harvest. ^g^There was a programming mistake in the computer-based questionnaire that caused all participants who reported working in only one farm to skip this question. ^h^Worked with someone who had COVID-19 symptoms or were known to be infected with SARS-CoV-2 since December 2019. ^i^Participants were asked whether they had experienced any of the following symptoms since December 2019: fever or chills, cough, shortness of breath, fatigue, myalgia, headache, new loss of sense of smell or taste, sore throat, blocked or runny nose, nausea or vomit, diarrhea, or any other symptom.

#### Mental health and economic impact of the COVID-19 pandemic

We first ran descriptive analyses of mental health and economic characteristics that have been previously reported as adversely affected by the COVID-19 pandemic (*n* = 395). We then fitted univariate binomial logistic regression models to examine the association of sociodemographic, household, occupational, and health-related characteristics with the overall impact of COVID-19 on farmworkers’ lives, regardless of their vaccination status (*n* = 334 participants with complete data). Lastly, we fitted a multivariable binomial logistic regression model with all characteristics with *p* < 0.20 in bivariate analyses, reduced our model, and estimated aRRs using the same approach described above.

All statistical analyses were conducted with R version 4.0.^1^

## Results

Most study participants were male (97.7%), were married or living as married (76.5%), lived in Tingambato (61.3%), and reported annual household earnings of less than $6,000 per year (66.6%) (Table 1), which was similar to the mean annual household income in rural Michoacan in 2020 (USD $5,800), but lower than the mean in 2022 (USD $7,000) (57). Participants had a median age of 37 years (interquartile range (IQR) 28–48) and lived with a median of 3 household members (IQR 2–5). Approximately half had completed middle school or had lower levels of education (56.4%), lived with other farmworkers (53.2%), and lacked health insurance (50.4%) (Table 1). Around three-quarters (77.5%) were overweight or obese, while a small percentage reported having diabetes (5.3%) or hypertension (11.1%). Notably, 61% of all participants reported experiencing symptoms indicative of SARS-CoV-2 infection since March 2020, yet only 3.8% had tested positive for COVID-19 (Table 1). Among farmworkers who experienced symptoms, approximately 40% continued working despite being symptomatic, while those who stayed home due to symptoms missed an average of 7.0 (SD 13.0) workdays.

At the time of the interview, 85.1% farmworkers were unvaccinated and, among these, 33.3% tested IgG positive (Table 2). Among the 56 (14.9%) participants who were partially or fully vaccinated, 87.5% tested IgG positive. Vaccinated farmworkers were older (mean age of 54.1 (SD 14.9) years) and more educated (50% had completed a bachelor’s degree or technical studies) compared to unvaccinated farmworkers (36.4 (12.3) years; and 13.7%, respectively) (Table 1). Additionally, vaccinated participants were more likely to be married or living as married (87.5%), reside in Tingambato (75.0%) or Uruapan (19.6%), and report household earnings greater than $6,000 per year (33.9%) compared to unvaccinated farmworkers (75, 67, 12.5%; respectively) (Table 1).

### Risk factors for positive SARS-CoV-2 result on IgG test among unvaccinated farmworkers

In bivariate analyses, we observed that farmworkers aged 60 years or older (58.3%) had a higher prevalence of IgG-positive SARS-CoV-2 infection than younger farmworkers (32.8% in those under age 30 and 32.5% in those aged 30–59 years; Table 2). Farmworkers living with children under age 5 (39.6%), with other farmworkers (39.8%), and/or in crowded housing (>2 persons/bedroom; 43.2%) also had higher seropositivity prevalence than their counterparts. Living with, but not working with, someone who had or may have had COVID-19 since the pandemic started (70.0%), having diabetes (63.6%), having had any SARS-CoV-2 infection symptoms since the pandemic started in December 2019 (36.1%), and having ever tested positive for COVID-19 (100.0%) were associated with a higher prevalence of IgG positivity. In contrast, farmworkers living outside of Tingambato or Uruapan (27.9%), cooking with a wood-burning stove (24.6%), and being rarely screened for either fever or COVID-19 symptoms upon arrival at work (30.0%) had a lower prevalence of IgG positivity than their counterparts (Table 2).

In multivariable analyses, we found that participants who lived with other farmworkers (aRR = 1.55; 95% CI: 1.05, 2.05), had ever lived with someone with COVID-19 (aRR = 1.82; 95% CI: 1.22, 2.43), or who had diabetes (aRR = 2.19; 95% CI: 1.53, 2.86) had a higher risk of IgG positivity (Table 2). Farmworkers who lived outside Tingambato or Uruapan (aRR = 0.71; 95% CI: 0.46, 0.96) or cooked with a wood-burning stove (aRR = 0.75; 95% CI: 0.55, 0.96) had a lower risk of having an IgG-positive test (Table 2).

### Mental health and economic impact of the COVID-19 pandemic among vaccinated and unvaccinated farmworkers

Of the 395 farmworkers enrolled in our study, 66.3% reported being somewhat or extremely negatively impacted by COVID-19, and 72.2% were moderately or very concerned about COVID-19 (Table 3). Notably, all nine female farmworkers reported been negatively impacted by COVID-19 (Table 4). About 9 and 11% of all farmworkers reported symptoms of depression and anxiety, respectively. Almost half (46.6%) reported decreased physical activity, whereas fewer (15.9%) reported increased substance use (Table 3). Approximately a quarter reported experiencing food insecurity (26.8%) or more difficulty paying bills (29.1%) since the COVID-19 pandemic started.

**Table 3:** Mental health and economic impact of COVID-19 pandemic among avocado farmworkers, Michoacan, Mexico, 2021 (*n* = 395).

	*n* (%)
COVID-19’s overall impact on life	
Not negative at all	72 (18.2)
Somewhat negative	183 (46.3)
Extremely negative	79 (20.0)
No answer	61 (15.4)
Concern about COVID-19	
Not or a little concerned	97 (24.5)
Moderately or very concerned	285 (72.2)
No answer	13 (3.3)
Mental health impact^a^	
Depression symptoms (≥2 on PHQ-2) in the 2 weeks preceding the study interview	
No	327 (82.8)
Yes	34 (8.6)
No answer	34 (8.6)
Anxiety symptoms (≥2 on GAD-2) in the 2 weeks preceding the study interview	
No	315 (79.8)
Yes	44 (11.1)
No answer	36 (9.1)
Felt unhappy with life	
No	330 (83.5)
Yes	54 (13.7)
No answer	11 (2.8)
Had more angry outbursts	
No	315 (79.8)
Yes	70 (17.7)
No answer	10 (2.5)
More arguing in household	
No	339 (85.8)
Yes	46 (11.7)
No answer	10 (2.5)
Had difficulty sleeping	
No	326 (82.5)
Yes	58 (14.7)
No answer	11 (2.8)
Less physical activity or exercise	
No	201 (50.9)
Yes	184 (46.6)
No answer	10 (2.5)
Overate/ate more unhealthy food	
No	287 (72.7)
Yes	93 (23.5)
No answer	15 (3.8)
Increased use of alcohol	
No	335 (84.8)
Yes	47 (11.9)
No answer	13 (3.3)
Increased use of tobacco	
No	360 (91.1)
Yes	23 (5.8)
No answer	12 (3.1)
Increased use of marijuana	
No	372 (94.2)
Yes	6 (1.5)
No answer	17 (4.3)
Increased use of other substances	
No	369 (93.4)
Yes	8 (2.0)
No answer	18 (4.6)
Increased use of any substance^b^	
No	332 (84.1)
Yes	63 (15.9)
No answer	0 (0.0)
Difficulty getting medical care or medications	
No	312 (79.0)
Yes	62 (15.7)
No answer	21 (5.32)
Loved one became sick or died of COVID-19	
No	255 (64.6)
Yes	128 (32.4)
No answer	12 (3.0)
Economic impact^c^	
Household food insecurity	
No (high or marginal security level)	289 (73.2)
Yes (low or very low security level)	106 (26.8)
No answer	0 (0.0)
More difficulty paying bills	
No	265 (67.1)
Yes	115 (29.1)
No answer	15 (3.8)
Received less money in remittances^d^	
No	9 (2.3)
Yes	13 (3.3)
Not applicable	373 (94.4)

GAD-2, Generalized Anxiety Disorder 2-item; PHQ-2, Patient Health Questionnaire 2-item. ^a^Unless specified, these outcomes indicate changes in feeling or behaviors compared to before the COVID-19 pandemic. ^b^Includes increased use of alcohol, tobacco products, marijuana, or other substances. ^c^These outcomes indicate changes since the COVID-19 pandemic started. ^d^This question was only asked to participants who were receiving remittances prior to the start of the COVID-19 pandemic.

**Table 4:** Sociodemographic, household, occupational, and health-related risk factors of COVID-19’s negative impact on life among avocado farmworkers, Michoacan, Mexico, May 2021 (*n* = 334).

Attribute	COVID-19’s overall impact on life			
	Somewhat or extremely negative (*n* = 262)	Not negative at all (*n* = 72)	*p*-value^a^	aRR (95% CI)^b^
Sex				
Female	9 (100.0)	0 (0.0)	0.04	--
Male	253 (77.8)	72 (22.2)		--
Age (years)				
<30	77 (80.2)	19 (19.8)	0.13	--
30–59	157 (80.5)	38 (19.5)		--
≥60	27 (65.9)	14 (34.1)		--
No answer	1 (50.0)	1 (50.0)		--
Education				
≤Primary school	57 (64.8)	31 (35.2)	<0.01	Reference
Middle school completed	72 (79.1)	19 (20.9)		1.18 (0.89, 1.48)
High school completed	59 (89.4)	7 (10.6)		1.31 (1.06, 1.57)
Bachelor’s degree or technical studies completed	66 (91.7)	6 (8.3)		1.33 (1.05, 1.60)
No answer	8 (47.1)	9 (52.9)		--
Marital status				
Not married or living as married	61 (81.3)	14 (18.7)	0.47	--
Married or living as married	200 (77.5)	58 (22.5)		--
No answer	1 (100.0)	0 (0.0)		--
Annual household income				
3,000	75 (75.0)	25 (25.0)	0.19	--
>3,000-6,000	97 (77.0)	29 (23.0)		--
>6,000	51 (86.4)	8 (13.6)		--
No answer	39 (79.6)	10 (20.4)		--
Community of residence				
Tingambato	168 (78.3)	46 (21.7)	0.33	--
Uruapan	28 (87.5)	4 (12.5)		
Other town^c^	68 (75.6)	22 (24.4)		--
Household size				
0–3	67 (77.9)	19 (22.1)	0.87	--
3–4	62 (80.5)	15 (19.5)		--
4–13	125 (77.6)	36 (22.4)		--
No answer	8 (80.0)	2 (20.0)		--
Children <18 y living in the home				
No	61 (73.5)	22 (26.5)	0.24	--
Yes	189 (79.7)	48 (20.3)		--
No answer	12 (85.7)	2 (14.3)		--
Children ≤5 y living in the home				
No	134 (74.9)	45 (25.1)	0.17	Reference
Yes	109 (81.3)	25 (18.7)		1.03 (0.97, 1.09)
No answer	19 (90.5)	2 (9.5)		
Living with other farmworkers				
No	115 (78.2)	32 (21.8)	0.98	--
Yes	139 (78.1)	39 (21.9)		--
No answer	8 (88.9)	1 (11.1)		--
Household crowding				
≤2 persons per bedroom	223 (79.6)	57 (20.4)	0.36	--
>2 persons per bedroom	27 (73.0)	10 (27.0)		--
No answer	12 (70.6)	5 (29.4)		--
Job title				
Owner	66 (81.5)	15 (18.5)	0.76	--
Full-time worker	84 (77.1)	25 (22.9)		--
Temporary worker^d^	8 (80.0)	2 (20.0)		--
No answer	104 (77.6)	30 (22.4)		--
Worked indoors				
No	84 (74.3)	29 (25.7)	0.23	--
Yes	173 (80.1)	43 (19.9)		--
No answer	5 (100.0)	0 (0.0)		--
Any COVID-19 related symptom since the pandemic started^e^				
No	87 (73.1)	32 (26.9)	0.08	Reference
Yes	175 (81.4)	40 (18.6)		1.04 (0.97, 1.11)
Self-reported positive COVID-19 test				
No	248 (77.7)	71 (22.3)	0.20	--
Yes	11 (91.7)	1 (8.3)		--
No answer	3 (100.0)	0 (0.0)		--
Vaccination status				
Not vaccinated	218 (77.9)	62 (22.1)	0.49	--
Partially or fully vaccinated^f^	12 (70.6)	5 (29.4)		--
No answer	32 (86.5)	5 (13.5)		--

Missing entries were excluded from bivariate analyses. ^a^*p*-values were calculated using univariate binomial logistic regression models. ^b^Adjusted RR and 95% CI were only obtained for variables with *p* < 0.20 in bivariate analyses and an importance value ≥ 0.5 in at least one of the 10 iterations in multivariable model. ^c^This category includes the following towns (distance to Tingambato): La Escondida (28.4 km), El Mesón (22.9 km), Angachuan (37.6 km), Pichátaro (55.4 km), Parangüitiro (15 km), Ziracuaretiro (18 km), San Angel Zurumucapio (23 km), Nahuatzen (45 km), Comachuén (45.2 km), Tancítaro (56.1 km), El Tarascón (Salvador Escalante 31.7 km), Nocutzepo (Erongarícuaro 49.2 km), Pátzcuaro (57.4 km). ^d^Includes workers who are hired for specific activities such as pesticide application and harvest. ^e^Participants were asked whether they had experienced any of the following symptoms since December 2019: fever or chills, cough, shortness of breath, fatigue, myalgia, headache, new loss of sense of smell or taste, sore throat, blocked or runny nose, nausea or vomit, diarrhea, or any other symptom. ^f^A farmworker was considered fully vaccinated if s/he had received all recommended doses of a COVID-19 vaccine that has been authorized or approved by the Mexican Federal Commission for Protection against Sanitary Risks (COFEPRIS) or is listed for emergency use by the World Health Organization (WHO). A farmworker was considered partially vaccinated if s/he had received at least one COFEPRIS-authorized COVID-19 vaccine dose but did not complete all recommended doses of that vaccine.

In bivariate analyses, we observed that a larger proportion of farmworkers under the age of 60 (80.2% of those under the age of 30 and 80.5% of those aged 30–59) reported being negatively impacted by COVID-19, compared to those aged 60 years or older (65.9%; Table 4). Farmworkers with an educational level higher than middle school (89.4% of those who had completed high school and 91.7% of those who had a bachelor’s degree or had completed technical studies), those with household earnings greater than $6,000 per year (86.4%), and those living with children under the age of 5 (81.3%) also reported being negatively impacted by COVID-19 more frequently than their counterparts. Lastly, in our bivariate analyses, we found that a larger proportion of farmworkers who had experienced any SARS-CoV-2 infection symptoms since the pandemic started (81.4%) or had ever tested positive for COVID-19 (91.7%) reported being negatively impacted by COVID-19, compared to farmworkers with no symptoms (73.1%) and no positive COVID-19 tests (77.7%; Table 4).

In multivariable analyses, we found that having completed high school (aRR = 1.31, 95% CI: 1.06, 1.57) or having a bachelor’s degree or technical studies (aRR = 1.33, 95% CI: 1.05, 1.60) was associated with a higher risk of reporting a negative impact of the COVID-19 pandemic (Table 4).

## Discussion

In this study of avocado farmworkers in Michoacan, no participants tested positive for current SARS-CoV-2 infection in May 2021, while 33% of unvaccinated farmworkers showed evidence of past infection (positive for IgG against SARS-CoV-2). This seroprevalence is slightly higher than the 25% noted among Guatemalan farmworkers from June 2020 to October 2021 (period that encompasses the first and second nationwide waves of new COVID-19 cases) (29), but well below the 53% observed among migrants in Tijuana, Mexico, from November 2020 to April 2021 (period that encompasses Baja California’s second wave of new COVID-19 cases) (58). Differences in SARS-CoV-2 seroprevalence between studies and populations must be interpreted with caution because of variations in timing of data collection, screening approaches, and between-country heterogeneity (59). Differences in routes of SARS-CoV-2 exposure could also play a role in the variability of seroprevalence estimates across studies.

In our study, we found that unvaccinated farmworkers who lived with other farmworkers, lived in more urban areas, and who had ever lived with someone with COVID-19 had a higher prevalence of IgG-positive SARS-CoV-2 infection. Similar findings, supporting the now well-established fact that household exposures substantially increase the risk of SARS-CoV-2 infection (33), were observed in a cross-sectional study of predominantly Mexican-born and low-income farmworkers in California (22). In Michoacan, we also found that farmworkers who cooked with a wood-burning stove had a lower risk of IgG positivity. This finding is unexpected, given previous research indicating that poor indoor air quality, particularly from indoor sources of air pollution such as cooking, may increase the risk of COVID-19 transmission and mortality (60). Nonetheless, it is possible that farmworkers with wood-burning stoves had better ventilation in their houses, built their stoves outside their houses, or spent more time outside than those with other stove types. We observed that livelihood with children under age 5 was associated with a higher seropositivity prevalence in our bivariate, but not multivariate, analyses. Mexico experienced one of the longest school closures globally due to the COVID-19 pandemic, lasting nearly 250 days from March to August 2020 (61). As farmworkers continued to work in-person during the pandemic, it is likely that their children had to be cared for by grandparents or other relatives while schools were closed, potentially exposing them to SARS-CoV-2 in environments other that their own.

Consistent with findings from other studies (30, 58, 62, 63), we observed an increased risk of IgG positivity among individuals with self-reported diabetes. However, only 3.3% of unvaccinated farmworkers in our study reported this medical condition, so this association must be interpreted with caution. The observed diabetes prevalence is lower than the 11.4% observed among primarily Mexican-born California farmworkers (30) and the 15.7% observed among the general Mexican population (21). Differences in diabetes prevalence could be partly explained by our exclusion of vaccinated farmworkers, among whom 17.9% reported diabetes and who were, on average, older (mean (SD): 54.1 (14.9) years) than those unvaccinated (36.4 (12.3) years). Nevertheless, differences could also be due to the significant proportion of unvaccinated farmworkers who lacked health insurance (54.6%) and might have undiagnosed medical conditions, such as diabetes. The mechanisms that explain the increased risk of SARS-CoV-2 infection and severity of COVID-19 among individuals with diabetes, including oxidative stress and the release of pro-inflammatory cytokines, have been widely documented (62, 64).

We observed that the impact of the COVID-19 pandemic among avocado farmworkers extended beyond infection. For example, 66% of farmworkers reported that their lives had been negatively affected by the pandemic. Notably, only a small number of participants reported depression (9%) and anxiety (11%) symptoms in the 2 weeks preceding the time of interview, which is lower than the prevalence reported in other Mexican populations since the COVID-19 pandemic started (65, 66). However, it is possible that farmworkers may have underreported their anxiety and depression symptoms (67, 68). We also observed that the COVID-19 pandemic had a substantial economic impact among vaccinated and unvaccinated farmworkers, as indicated by their reduced ability to pay bills and food insecurity. The prevalence of food insecurity among Michoacan farmworkers (27%) is lower than the 37% observed among California farmworkers in November 2020 (33). The proportion of Michoacan farmworkers who experienced more difficulties paying bills since the COVID-19 pandemic started (29%) is in line with the 28% observed among California farmworkers in November 2020 (33), but much lower than the 62% noted in a statewide study of farmworkers in California conducted between July 2021 and April 2022 (34). Nonetheless, it is essential to recognize that comparing the experiences of farmworkers across multiple countries may not provide a reliable basis for assessment due to the significant differences in their socio-economic conditions, healthcare systems, and governmental support mechanisms.

To our knowledge, our study is the first to determine the prevalence of SARS-CoV-2 infection among Mexican farmworkers and to examine the impact of the COVID-19 pandemic in this vulnerable population. However, several limitations should be considered. Our analysis is cross-sectional, and our sample size is modest but consistent with figures from studies in other countries (27, 69, 70). In addition, our sample was not random, which limits the generalizability of our results to the larger Mexican farmworker population and rural communities. Moreover, waning antibodies, especially among individuals who experienced mild or asymptomatic infection, may have contributed to misclassification in IgG positivity for individuals infected early in the COVID-19 pandemic (71–73). Finally, our survey was self-administered, which may have led to incomplete or inaccurate data, particularly among farmworkers with lower educational levels.

Our findings indicate that avocado farmworkers in Michoacan have been significantly affected by the COVID-19 pandemic, both in terms of morbidity and its economic impact on their lives. Given farmworkers’ risk of repeated SARS-CoV-2 infections due to their living conditions, poor access to health care, and high prevalence of comorbidities (e.g., obesity, diabetes) that increase the risk of severe COVID-19 and long COVID (74, 75), a multi-faceted approach is required to address their unique needs and challenges. Potential strategies for supporting farmworkers may include increasing their access to healthcare (e.g., expanding telemedicine options and mobile clinics) and providing financial and mental health support, especially for farmworkers who are unable to work due to COVID-19 or long COVID symptoms.

## Data availability statement

The raw data supporting the conclusions of this article will be made available by the authors, without undue reservation.

## Ethics statement

The studies involving humans were approved by the Institutional Review Boards at University of California, Berkeley and Escuela Nacional de Estudios Superiores Unidad Morelia, Universidad Nacional Autónoma de México (UNAM). The participants provided their written informed consent to participate in this study.

## Author contributions

CA-A and AM had full access to all the data in the study and take responsibility for the integrity of the data and the accuracy of the data analysis. CA-A, MT-O, FC, AS-V, JD, BE, and AM: concept and design. CA-A, MT-O, FM-A, MR-B, DB-S, FC, AS-V, DL-C, JD, BE, and AM: acquisition, analysis, or interpretation of data. CA-A, MT-O, and AM: drafting of the manuscript. CA-A, MT-O, FM-A, MR-B, DB-S, FC, AS-V, DL-C, JD, BE, and AM: critical revision of the manuscript for important intellectual content. CA-A, FM-A, and AM: statistical analysis. CA-A, FC, AS-V, DL-C, JD, BE, and AM: obtained funding. CA-A, MT-O, MR-B, DB-S, BE, and AM: administrative, technical, or material support. CA-A and AM: supervision. All authors contributed to the article and approved the submitted version.

## Footnotes

^1^R Project for Statistical Computing, https://www.r-project.org.

## References

[1] Secretaría de Salud. *Se confirma en México caso importado de coronavirus COVID-19*. (2020). Available at: http://www.gob.mx/salud/prensa/077-se-confirma-en-mexico-caso-importado-de-coronavirus-covid-19. (Accessed Oct 20, 2023).

[2] Secretaría de Salud. *Comunicado Técnico Diario COVID-19*. (2020). Available at: https://www.gob.mx/cms/uploads/attachment/file/544174/Comunicado_Tecnico_Diario_COVID-19_2020.03.28.pdf.

[3] Gobierno de México. *Jornada Nacional de Sana Distancia*. (2020). Available at: https://www.gob.mx/cms/uploads/attachment/file/541687/Jornada_Nacional_de_Sana_Distancia.pdf.

[4] Knaul FM Touchton M Arreola-Ornelas H Atun R Anyosa RJC Frenk J. Punt politics as failure of health system stewardship: evidence from the COVID-19 pandemic response in Brazil and Mexico. Lancet Reg Health Am. (2021) 4:100086. doi: 10.1016/j.lana.2021.100086 34664040 PMC8514423

[5] Diario Oficial de la Federación. *Emergencia Sanitaria generada por el virus SARS-CoV-2 (Decreto del 31 de marzo de 2020)*. (2020). Available at: https://www.dof.gob.mx/nota_detalle.php?codigo=5590914&fecha=31/03/2020#gsc.tab=0.

[6] Gobierno de México. *COVID-19 Medidas Economicas. 2020. Nueva normalidad. Reactivación de la economía mexicana de forma responsable y segura*. Available at: http://www.gob.mx/covid19medidaseconomicas/acciones-y-programas/nueva-normalidad-244196.

[7] Organización Panamericana de la Salud. *Actualización Epidemiológica: Enfermedad por coronavirus (COVID-19)*. (2020). Available at: https://www.paho.org/es/documentos/actualizacion-epidemiologica-enfermedad-por-coronavirus-covid-19-26-agosto-2020.

[8] Sánchez-Talanquer M González-Pier E Sepúlveda J Abascal-Miguel L Fieldhouse J Del Río C. *Mexico’s response to COVID-19: A case study*. UCSF Institute for Global Health Sciences. (2021). Available at: https://globalhealthsciences.ucsf.edu/sites/globalhealthsciences.ucsf.edu/files/mexico-covid-19-case-study-english.pdf.

[9] Instituto Nacional de Estadística y Geografía. *Población rural y urbana*. Cuéntame de México. (2020). Available at: https://cuentame.inegi.org.mx/poblacion/rur_urb.aspx?tema=P. (Accessed Oct 21, 2023).

[10] Secretaria de agricultura y desarrollo rural (SADER), Servicio de Información Agroalimentaria y Pesquera (SIAP). *Empleo en el sector agropecuario y pesquero*. (2022). Available at: https://www.gob.mx/cms/uploads/attachment/file/729352/An_lisis_de_Empleo_en_Actividades_agropecuarias_y_pesqueras_1_Trim_2022.pdf. (Accessed Oct 21, 2023).

[11] International Fund for Agricultural Development (IFAD). *Investing in rural people in Mexico*. (2017). Available at: https://www.ifad.org/en/web/knowledge/-/invirtiendo-en-la-poblaci%C3%B3n-rural-en-m%C3%A9xico. (Accessed Oct 20, 2023).

[12] Ortiz-Hernández L Pérez-Sastré MA. Inequidades sociales en la progresión de la COVID-19 en población mexicana. Rev Panam Salud Pública. (2020) 44:1. doi: 10.26633/RPSP.2020.106 PMC7505478 32983234

[13] Rivera-Hernandez M Ferdows NB Kumar A. The impact of the COVID-19 epidemic on older adults in rural and urban areas in Mexico. Carr D, editor. J Gerontol Ser B. (2021) 76:e268–74. doi: 10.1093/geronb/gbaa227, PMID: 33367752 PMC7798580

[14] Cohen JH Mitchel AP Montiel Ishino FA. Evaluating the indigenous response to COVID-19 in rural Oaxaca, Mexico. J Glob Health. (2023) 13:51. doi: 10.7189/jogh.13.03051, PMID: 37792892 PMC10550196

[15] Chávez-Almazán LA Díaz-González L Rosales-Rivera M. Determinantes socioeconómicos de salud y COVID-19 en México. Gac Méd México. (2022) 158:6662. doi: 10.24875/GMM.21000302 35404927

[16] Jensen N Kelly AH Avendano M. The COVID-19 pandemic underscores the need for an equity-focused global health agenda. Hum Soc Sci Commun. (2021) 8:1–6. doi: 10.1057/s41599-020-00700-x

[17] Flores-Ramírez R Berumen-Rodríguez AA Martínez-Castillo MA Alcántara-Quintana LE Díaz-Barriga F De L-MLD. A review of environmental risks and vulnerability factors of indigenous populations from Latin America and the Caribbean in the face of the COVID-19. Glob Public Health. (2021) 16:975–99. doi: 10.1080/17441692.2021.1923777, PMID: 33966608

[18] Lomelí EV Molina MEJ. *Brechas de acceso a la salud en México en el marco de la nueva ruralidad*. Ciudad de México: Comisión Económica para América Latina y el Caribe (CEPAL), p. 128. (2023). Available at: https://www.cepal.org/es/publicaciones/48750-brechas-acceso-la-salud-mexico-marco-la-nueva-ruralidad.

[19] Consejo Nacional de Evaluación de la Política de Desarrollo Social. *Líneas de Pobreza por Ingresos*. (2022). Available at: https://www.coneval.org.mx/Medicion/Documents/Lineas_de_Pobreza_por_Ingresos/Lineas_de_Pobreza_por_Ingresos_COVID_feb_2022.pdf. (Accessed Oct 21, 2023).

[20] Carnalla M Basto-Abreu A Stern D Bautista-Arredondo S Shamah-Levy T Alpuche-Aranda CM. Acceptance, refusal, and hesitancy of Covid-19 vaccination in Mexico: Ensanut 2020 Covid-19. Salud Publica Mex. (2021) 63:598–606. doi: 10.21149/12696, PMID: 35099875

[21] Basto-Abreu AC López-Olmedo N Rojas-Martínez R Aguilar-Salinas CA La Cruz-Góngora VVD Rivera-Dommarco J. Prevalence of diabetes and glycemic control in Mexico: national results from 2018 and 2020. Salud Publica Mex. (2021) 63:725–33. doi: 10.21149/12842, PMID: 35099912

[22] Lewnard JA Mora AM Nkwocha O Kogut K Rauch SA Morga N. Prevalence and clinical profile of severe acute respiratory syndrome coronavirus 2 infection among farmworkers, California, USA, June-November 2020. Emerg Infect Dis. (2021) 27:1330–42. doi: 10.3201/eid2705.204949, PMID: 33657340 PMC8084509

[23] Chen YH Glymour M Riley A Balmes J Duchowny K Harrison R. Excess mortality associated with the COVID-19 pandemic among Californians 18–65 years of age, by occupational sector and occupation: march through November 2020. PLoS One. (2021) 16:e0252454. doi: 10.1371/journal.pone.0252454, PMID: 34086762 PMC8177528

[24] Chen YH Riley AR Duchowny KA Aschmann HE Chen R Kiang MV. COVID-19 mortality, and excess mortality among working-age residents in California, USA, by occupational sector: a longitudinal cohort analysis of mortality surveillance data. Lancet Public Health. (2022) 7:e744–53. doi: 10.1016/S2468-2667(22)00191-8, PMID: 36057273 PMC9433054

[25] Riley AR Chen YH Matthay EC Glymour MM Torres JM Fernandez A. Excess mortality among Latino people in California during the COVID-19 pandemic. SSM Popul Health. (2021) 15:100860. doi: 10.1016/j.ssmph.2021.100860 34307826 PMC8283318

[26] California Institute for Rural Studies. *Instituto de Estudios Rurales de California*. COVID-19 Farmworker Study (COFS). (2021). Available at: https://cirsinc.org/es/covid-19-farmworker-study/. (Accessed Oct 20, 2023).

[27] Rose DC Shortland F Hall J Hurley P Little R Nye C. The impact of COVID-19 on farmers’ mental health: a case study of the UK. J Agromedicine. (2022) 28:346–64. doi: 10.1080/1059924X.2022.2137616 36263759

[28] Meredith D McNamara J Van Doorn D Richardson N. Essential and vulnerable: implications of Covid-19 for farmers in Ireland. J Agromedicine. (2020) 25:357–61. doi: 10.1080/1059924X.2020.1814920, PMID: 32921287

[29] Olson D Calvimontes DM Lamb MM Guzman G Barrios E Chacon A. Clinical and economic impact of COVID-19 on agricultural workers, Guatemala. Emerg Infect Dis. (2022) 28:S277–87. doi: 10.3201/eid2813.212303, PMID: 36502430 PMC9745239

[30] Mora AM Lewnard JA Kogut K Rauch SA Hernandez S Wong MP. Risk factors associated with SARS-CoV-2 infection among farmworkers in Monterey County, California. JAMA Netw Open. (2021) 4:e2124116. doi: 10.1001/jamanetworkopen.2021.24116, PMID: 34524438 PMC8444020

[31] Kissam E. The impact of the COVID-19 pandemic on California farmworkers: better local data collection and reporting will improve strategic response. Stat J IAOS. (2020) 36:867–98. doi: 10.3233/SJI-200763

[32] Flores Mariscal J. *El trabajo jornalero agricola sus condiciones de precariedad en mexico y experiencias en la region latinoamericana para mejorar su acceso a la seguridad social*. Ciudad de México: Seguridad Social para el Bienestar. (Conferencia Interamericana de Seguridad Social). (2022). Available at: https://ciss-bienestar.org/cuadernos/pdf/el-trabajo-jornalero-agricola-sus-condiciones-de-precariedad-en-mexico-y-experiencias-en-la-region-latinoamericana-para-mejorar-su-acceso-a-la-seguridad-social.pdf. (Accessed Oct 20, 2023).

[33] Mora AM Lewnard JA Rauch S Kogut K Jewell N Cuevas M. Impact of COVID-19 pandemic on California farmworkers’ mental health and food security. J Agromedicine. (2022) 27:303–14. doi: 10.1080/1059924X.2022.2058664, PMID: 35333134

[34] Brown P Flores E Padilla A. *Farmworker health in California: health in a time of contagion, drought, and climate change*. University of California Merced (2022). Available at: https://clc.ucmerced.edu/sites/clc.ucmerced.edu/files/page/documents/fwhs_report_2.2.2383.pdf.

[35] National Center for Farmworkers Health N. *Farmworkers COVID-19 community assessments: Yakima County, WA*. (2022). Available at: http://www.ncfh.org/uploads/3/8/6/8/38685499/yakima_wa_rapid_assessment_-_survey_report_2022.pdf.

[36] Gordean RS. The avocado war, from “Alligator’s pear” to “green gold” (2021) 26:68–75.

[37] Servicio de Información Agroalimentaria y Pesquera (SIAP). Avance de Siembras y Cosechas. Valuación Profesional Mexicana. (2022). Available at: https://nube.siap.gob.mx/avance_agricola/.

[38] Avocado Institute of Mexico. *Who is APEAM? Avocado Institute of Mexico*. (2020). Available at: https://avocadoinstitute.org/apeam-mhaia/about-apeam/. (Accessed Oct 20, 2023).

[39] Consejo Estatal de Población del Estado de Michoacán. *Población en Michoacán 2020*. Gobierno de Michoacán (2021). Available at: https://coespo.michoacan.gob.mx/wp-content/uploads/2021/02/Poblacion-en-Michoacan-2020.pdf.

[40] Bada X Feldmann AE. *Mexico’s Michoacán state: mixed migration flows and transnational links*. Forced Migration Review (2017). Available at: https://www.fmreview.org/latinamerica-caribbean/bada-feldmann.

[41] Organización Internacional para las Migraciones (OIM), Estados Unidos Mexicanos (México). *Perfil 2022: Indicadores de Gobernanza de la Migración a Nivel Local, Estado de Michoacán*. Ginebra: Organización Internacional para las Migraciones (OIM). (2022). Available at: https://mexico.iom.int/sites/g/files/tmzbdl1686/files/documents/MGI%20RESUMEN%20EJECUTIVO%20MICHOACAN.pdf.

[42] Instituto Nacional de Estadística y Geografía (INEGI). *Censo de Población y Vivienda (2020). Panorama sociodemográfico de Michoacán de Ocampo*. (2021).

[43] Servicio Nacional de Sanidad, Inocuidad y Calidad Agroalimentaria S. *Aguacate michoacano, oro verde para los pequeños productores*. (2021). Available at: http://www.gob.mx/senasica/articulos/aguacate-michoacano-oro-verde-para-los-pequenos-productores.

[44] Zepeda AV Limón MLS. “Oro verde”, educación e inseguridad en ciudades medias: Ciudad Guzmán y Uruapan, México. Rev Cienc Soc Ve. (2020) 26:414–25.

[45] Ortiz-Paniagua CF. Agricultura y economía municipal en Michoacán desde una perspectiva de vulnerabilidad/agriculture and municipality economics in Michoacan from a perspective of vulnerability. CIBA Rev Iberoam Las Cienc Biológicas Agropecu. (2017) 6:63–91. doi: 10.23913/ciba.v6i12.69

[46] Ortiz-Paniagua CF Zamora-Torres AI Bonales-Valencia J Ortiz-Paniagua CF Zamora-Torres AI Bonales-Valencia J. Vulnerabilidad económica municipal del impacto agrícola ante condiciones de cambio climático en Michoacán. Anál Econ. (2018) 33:73–93. doi: 10.24275/uam/azc/dcsh/ae/2018v33n82/Ortiz

[47] Asociación de Productores y Empacadores de aguacate de Michoacán. *Juntas Locales de Sanidad Vegetal*. (2022). Available at: https://www.apeamac.com/juntas-locales-de-sanidad-vegetal/.

[48] Gobierno de México. *Política nacional de vacunación contra el virus SARS-CoV-2, para la prevención de la COVID-19 en México*. (2021). Available at: https://coronavirus.gob.mx/wp-content/uploads/2021/01/PolVx_COVID_-11Ene2021.pdf.

[49] Barros-Sierra D Zepeda-Tello R Tamayo-Ortiz M Gutiérrez-Díaz HO Pérez-Chávez VA Rosa-Parra JA. SARS-CoV-2 seroprevalence and respiratory disease disability claims in Mexico City metropolitan area. Salud Publica Mex. (2023) 65:334–43. doi: 10.21149/14545 38060902

[50] Kroenke K Spitzer RL Williams JBW. The patient health Questionnaire-2: validity of a two-item depression screener. Med Care. (2003) 41:1284–92. doi: 10.1097/01.MLR.0000093487.78664.3C 14583691

[51] Kroenke K Spitzer RL Williams JBW Monahan PO Löwe B. Anxiety disorders in primary care: prevalence, impairment, comorbidity, and detection. Ann Intern Med. (2007) 146:317–25. doi: 10.7326/0003-4819-146-5-200703060-00004, PMID: 17339617

[52] USDA. *U.S. household food security survey module: six-item short form economic research service*. Economic Research Service (2012). Available at: https://www.ers.usda.gov/media/8271/hh2012.pdf.

[53] *Elecsys anti-SARS-CoV-2 S*. (2022). Available at: https://www.fda.gov/media/144037/download.

[54] Food and Agriculture Organization of the United Nations (FAO). *AdviseDx SARS-CoV-2 IgG II*. (2022). Available at: https://www.fda.gov/media/146371/download.

[55] Buuren SV Groothuis-Oudshoorn K. Mice: multivariate imputation by chained equations in *R*. J Stat Softw. (2011) 45. doi: 10.18637/jss.v045.i03

[56] Lee Y. *LogisticRR: Adjusted relative risk from logistic regression*. (2020). Available at: https://cran.r-project.org/web/packages/logisticRR/index.html. (Accessed May 16, 2023).

[57] Instituto Nacional de Estadística y Geografía (INEGI). *Encuesta Nacional de Ingresos y Gastos de los Hogares 2022 (ENIGH), Michoacán de Ocampo*. (2022). Available at: https://www.inegi.org.mx/contenidos/programas/enigh/nc/2022/doc/enigh2022_ns_presentacion_resultados_mich.pdf. (Accessed Nov 18, 2023).

[58] Bojorquez I Strathdee S Garfein R Benson C Chaillon A Ignacio C. The impact of the COVID-19 pandemic among migrants in shelters in Tijuana, Baja California. Mexico BMJ Glob Health. (2022) 7:e007202. doi: 10.1136/bmjgh-2021-007202, PMID: 35277428 PMC8919131

[59] Axfors C Pezzullo AM Contopoulos-Ioannidis DG Apostolatos A Ioannidis JP. Differential COVID-19 infection rates in children, adults, and elderly: systematic review and meta-analysis of 38 pre-vaccination national seroprevalence studies. J Glob Health. (2023) 13:06004. doi: 10.7189/jogh.13.06004, PMID: 36655924 PMC9850866

[60] Bourdrel T Annesi-Maesano I Alahmad B Maesano CN Bind MA. The impact of outdoor air pollution on COVID-19: a review of evidence from in vitro, animal, and human studies. Eur Respir Rev. (2021) 30:200242. doi: 10.1183/16000617.0242-2020 33568525 PMC7879496

[61] Organization for Economic, Co-operation and Development. *The state of global education: 18 months into the pandemic*. OCDE (2021). Available at: https://read.oecd-ilibrary.org/education/the-state-of-global-education_1a23bb23-en.

[62] Sen S Chakraborty R Kalita P Pathak MP. Diabetes mellitus and COVID-19: understanding the association in light of current evidence. World J Clin Cases. (2021) 9:8327–39. doi: 10.12998/wjcc.v9.i28.8327, PMID: 34754842 PMC8554438

[63] Eskenazi B Rauch S Iurlaro E Gunier RB Rego A Gravett MG. Diabetes mellitus, maternal adiposity, and insulin-dependent gestational diabetes are associated with COVID-19 in pregnancy: the INTERCOVID study. Am J Obstet Gynecol. (2022) 227:74.e1–74.e16. doi: 10.1016/j.ajog.2021.12.032, PMID: 34942154 PMC8686449

[64] Steenblock C Schwarz PEH Ludwig B Linkermann A Zimmet P Kulebyakin K. COVID-19 and metabolic disease: mechanisms and clinical management. Lancet Diabetes Endocrinol. (2021) 9:786–98. doi: 10.1016/S2213-8587(21)00244-8, PMID: 34619105 PMC8489878

[65] Hernández-Díaz Y Genis-Mendoza AD Ramos-Méndez MÁ Juárez-Rojop IE Tovilla-Zárate CA González-Castro TB. Mental health impact of the COVID-19 pandemic on Mexican population: a systematic review. Int J Environ Res Public Health. (2022) 19:6953. doi: 10.3390/ijerph19116953, PMID: 35682536 PMC9180045

[66] Valencia PD Torres-Quispe MA Sánchez-Cayo S Reyes-Aguilar RF Acevedo-Cahuana AG. Factors associated with depressive symptomatology during the COVID-19 pandemic in Mexico: a 2021 national survey. J Affect Disord. (2022) 317:212–20. doi: 10.1016/j.jad.2022.08.088, PMID: 36041583 PMC9419429

[67] Ferris-Day P Hoare K Wilson RL Minton C Donaldson A. An integrated review of the barriers and facilitators for accessing and engaging with mental health in a rural setting. Int J Ment Health Nurs. (2021) 30:1525–38. doi: 10.1111/inm.12929, PMID: 34482621

[68] Oliveira JL d Almeida JCP d Pauli AJC d Moitinho MR Fiorati RC Souza J d. Psychosocial impacts of the COVID-19 pandemic among settled women: a longitudinal study. Rev Lat Am Enfermagem. (2023) 31:e3831. doi: 10.1590/1518-8345.6123.3832 36888794 PMC9991003

[69] Quandt SA LaMonto NJ Mora DC Talton JW Laurienti PJ Arcury TA. COVID-19 pandemic among Latinx farmworker and nonfarmworker families in North Carolina: knowledge, risk perceptions, and preventive behaviors. Int J Environ Res Public Health. (2020) 17:5786. doi: 10.3390/ijerph17165786, PMID: 32785108 PMC7459606

[70] Keeney AJ Quandt A Villaseñor MD Flores D Flores L. Occupational stressors and access to COVID-19 resources among commuting and residential Hispanic/Latino farmworkers in a US-Mexico border region. Int J Environ Res Public Health. (2022) 19:763. doi: 10.3390/ijerph19020763, PMID: 35055585 PMC8775392

[71] Alfego D Sullivan A Poirier B Williams J Grover A Gillim L. A population-based analysis of the longevity of SARS-CoV-2 antibody seropositivity in the United States. EClinicalMedicine. (2021) 36:902. doi: 10.1016/j.eclinm.2021.100902 PMC8143650 34056568

[72] Carvalho Á Henriques AR Queirós P Rodrigues J Mendonça N Rodrigues AM. Persistence of IgG COVID-19 antibodies: a longitudinal analysis. Front Public Health. (2023) 10:1069898. doi: 10.3389/fpubh.2022.1069898, PMID: 36703818 PMC9872107

[73] Liu G Rusling JF. COVID-19 antibody tests and their limitations. ACS Sens. (2021) 6:593–612. doi: 10.1021/acssensors.0c02621 33544999

[74] Farmworker Justice. *Long COVID: Farmworker rights and protections*. (2022). Available at: https://www.farmworkerjustice.org/wp-content/uploads/2022/09/Long-COVID-ISSUE-BRIEF.pdf.

[75] Mora AM Kogut K Sandhu NK Ridgway D Patty CM Renteria M. SARS-CoV-2 infection and long COVID among California farmworkers. J Rural Health. (2023) 2023:1–11. doi: 10.1111/jrh.12796 37715721

