# Supplementary material for: Prevalence of SARS-CoV-2 infection and impact of the COVID-19 pandemic in avocado farmworkers from Mexico
Source: Front Public Health. 2023 Dec 20;11:1252530. doi: 10.3389/fpubh.2023.1252530 (PMC10761533; doi:10.3389/fpubh.2023.1252530)

SUPPLEMENTARY MATERIAL

Prevalence of SARS-CoV-2 infection and impact of the COVID-19 pandemic in avocado farmworkers from Mexico

Cynthia Armendáriz-Arnez[1†], Marcela Tamayo-Ortiz[2], Francisco Mora-Ardila[3], María Esther Rodríguez-Barrena[1], David Barros-Sierra[4], Federico Castillo[5], Armando Sánchez-Vargas[6], David Lopez-Carr[7], Julianna Deardorff[8], Brenda Eskenazi[8], Ana M. Mora[8†]

[1]Escuela Nacional de Estudios Superiores (ENES) Unidad Morelia, Universidad Nacional Autónoma de México (UNAM), Morelia, Mexico

[2]Instituto Mexicano del Seguro Social (IMSS), Mexico City, Mexico

[3]Instituto de Investigaciones en Ecosistemas y Sustentabilidad, Universidad Nacional Autónoma de México (UNAM), Morelia, Mexico

[4]Department of Environmental Science, Policy and Management, University of California, Berkeley, Berkeley, CA, United States

[5]Institute of Economic Research, Universidad Nacional Autónoma de Mexico (UNAM), Mexico City, Mexico

[6]Department of Geography, University of California, Santa Barbara, Santa Barbara, CA, United States

[7]Center for Environmental Research and Community Health (CERCH), School of Public Health, University of California, Berkeley, Berkeley, CA, United States

[†]Contributed equally to this work

Figure S1. COVID-19 epidemic curve in Michoacan, Mexico, between April 2020 and March 2022. Adapted from: COVID-19 Mexico. [Gobierno de Mexico]. https://datos.covid-19.conacyt.mx/#DOView. Accessed October 19, 2023.

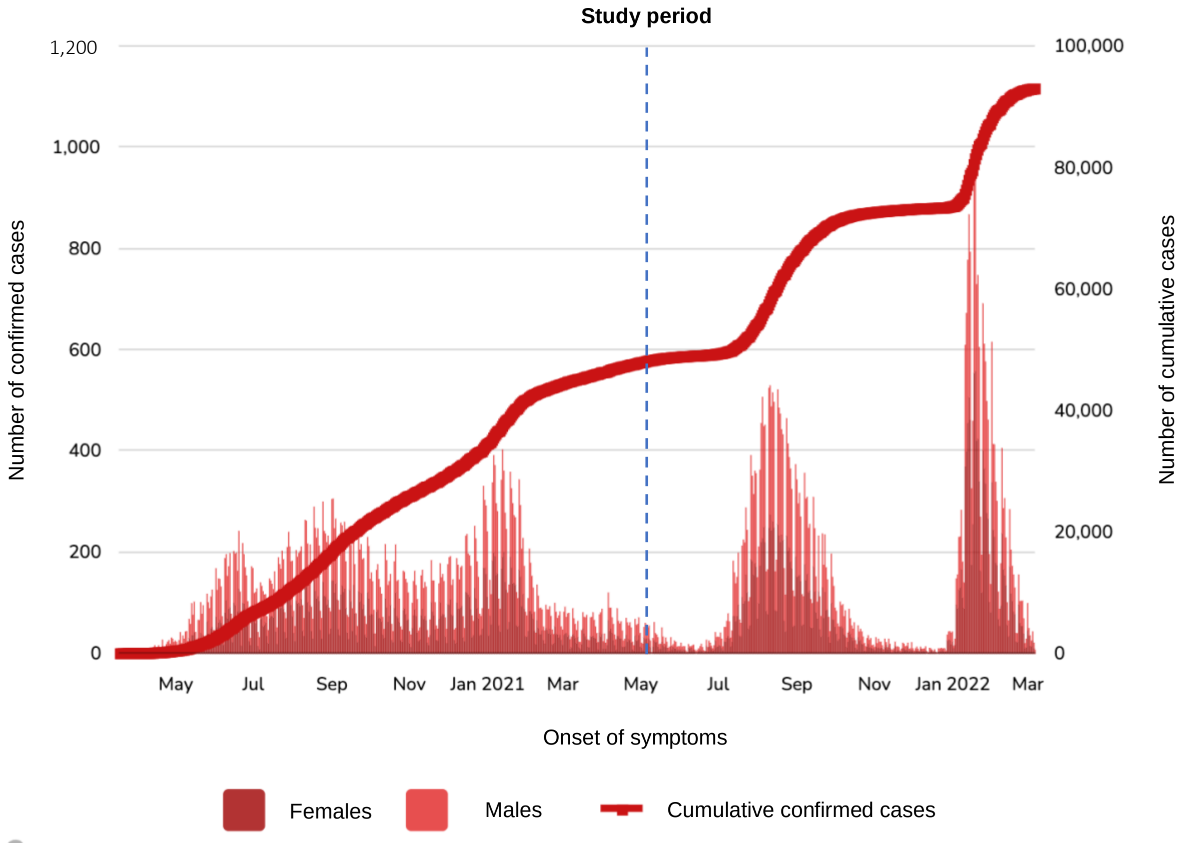

Supplement: Supplementary file 1 [file Image_1.PDF]
